# Hypertriglyceridaemia-Associated Acute Pancreatitis: Risk Stratification, Drivers, and Prevention of Recurrence

**DOI:** 10.3390/diseases14020047

**Published:** 2026-01-30

**Authors:** Federica Fogacci, Arrigo F. G. Cicero

**Affiliations:** 1 Hypertension and Cardiovascular Risk Factors Research Centre, Medical and Surgical Sciences Department, Alma Mater Studiorum University of Bologna, 40100 Bologna, Italy; federica.fogacci@studio.unibo.it; 2 Department of Medical Pharmacology, Medical Faculty, Ataturk University, Erzurum 25240, Turkey; 3 Cardiovascular Medicine Unit, IRCCS Azienda Ospedaliero–Universitaria di Bologna, 40100 Bologna, Italy

**Keywords:** acute pancreatitis, hypertriglyceridaemia, chylomicronaemia, familial chylomicronaemia syndrome, multifactorial chylomicronaemia, apolipoprotein C-III, triglyceride-rich lipoproteins, recurrence prevention

## Abstract

Hypertriglyceridaemia is the third most common aetiology of acute pancreatitis and a leading cause of recurrence in specialized lipid clinics. The risk of acute pancreatitis rises steeply once triglycerides exceed approximately 10 mmol/L (≈885 mg/dL). Still, clinically meaningful risk may occur at lower levels in the presence of chylomicronaemia, metabolic stress, or pregnancy. This mini-review synthesizes contemporary evidence on epidemiology, mechanistic links between triglyceride-rich lipoproteins and pancreatic injury, and the practical distinction between secondary (acquired) and genetic drivers of severe hypertriglyceridaemia. We summarize acute management strategies aimed at rapid triglyceride reduction (including insulin-based approaches and therapeutic plasma exchange in selected scenarios) and focus on long-term prevention of recurrence through lifestyle interventions, correction of secondary contributors, and triglyceride-lowering pharmacotherapy. Finally, we discuss emerging RNA-targeted therapies against apolipoprotein C-III and angiopoietin-like 3, which are reshaping prevention strategies for familial and persistent chylomicronaemia and may reduce pancreatitis burden in the highest-risk phenotypes.

## 1. Introduction

Hypertriglyceridaemia-associated acute pancreatitis (HTG-AP) is an increasingly recognised entity, linked to the rising prevalence of obesity, type 2 diabetes, and alcohol use. Across cohorts, HTG-AP accounts for roughly 2–10% of acute pancreatitis presentations but may be over-represented in younger patients and in recurrent disease [1,2,3]. Because triglyceride levels can fall rapidly with fasting and intravenous fluids, early sampling is essential, and chylomicronaemia should be suspected when serum appears lactescent. Clinically, the pancreatitis risk rises sharply once triglycerides exceed ~10 mmol/L (~885 mg/dL) and becomes particularly high above 20 mmol/L (~1770 mg/dL), especially in the presence of ‘second hits’ such as uncontrolled diabetes, pregnancy, alcohol, or certain medicines [4,5]. Current international pancreatitis guidance emphasizes supportive care, while triglyceride-specific interventions remain variably adopted due to limited randomized evidence [6,7]. This review summarizes risk epidemiology, mechanistic pathways, and a pragmatic approach to identifying secondary and genetic drivers of severe hypertriglyceridaemia, with a focus on preventing pancreatitis recurrence.

## 2. Materials and Methods

This narrative review was developed to support clinical decision-making in severe hypertriglyceridaemia and suspected HTG-AP. We searched PubMed and major society/regulatory websites (December 2010–December 2025) for English-language evidence using combinations of the terms ‘hypertriglyceridemia’, ‘chylomicronemia’, ‘familial chylomicronemia’, ‘acute pancreatitis’, ‘plasmapheresis/therapeutic plasma exchange’, ‘insulin’, ‘apolipoprotein C-III’, ‘ANGPTL3’, ‘olezarsen’, ‘volanesorsen’, and ‘plozasiran’. Priority was given to international guidelines, randomized trials, meta-analyses, and large observational cohorts; mechanistic studies were included when they clarified biological plausibility. Reference lists of eligible papers were hand-searched to identify additional key sources.

## 3. Epidemiology and Risk Gradient

Acute pancreatitis incidence varies widely by geography and case-mix, with contemporary global datasets highlighting substantial heterogeneity in aetiology and outcomes. Hypertriglyceridaemia is typically considered causative when triglycerides are ≥11.3 mmol/L (≥1000 mg/dL) and no alternative dominant aetiology is found, although some clinicians use a lower threshold (~10 mmol/L) when chylomicronaemia is evident [1,3]. Large population studies demonstrate a graded relationship between triglycerides and pancreatitis, with risk detectable even in the 2–10 mmol/L range in susceptible individuals and with the steepest rise at higher concentrations. In a Danish cohort, nonfasting triglycerides of 2.0–2.9 mmol/L were associated with a hazard ratio (HR) of 2.3 (95% CI 1.3–4.0), while triglycerides ≥5.0 mmol/L were associated with an HR of 8.7 (4.9–15.5), corresponding to ~7 and 12 events per 10,000 person-years, respectively. Among hospitalised HTG-AP cohorts, recurrent pancreatitis is common, reflecting persistent or recurrent severe hypertriglyceridaemia and incomplete correction of secondary contributors [6,7].

Importantly, triglycerides may be a ‘marker and mediator’: they can reflect broader metabolic inflammation and simultaneously contribute directly to pancreatic lipotoxicity. Thus, risk assessment should integrate both the absolute triglyceride level and the clinical context (fasting status, glycaemic control, alcohol, pregnancy, renal function, and medications) [6,7].

## 4. Pathophysiological Links Between Triglyceride-Rich Lipoproteins and Pancreatic Injury

Severe hypertriglyceridaemia is usually characterized by circulating chylomicrons, with or without very-low-density lipoproteins (VLDL). Chylomicrons increase plasma viscosity and may impair pancreatic microcirculation, predisposing to local ischaemia. Within the inflamed pancreas, hydrolysis of triglyceride-rich lipoproteins by pancreatic lipase generates high local concentrations of unbound free-fatty acids, which can overwhelm albumin buffering, disrupt acinar and endothelial membranes, and amplify inflammation and microthrombosis. Mechanistic models support a central role for lipase-driven lipotoxicity in amplifying local and systemic inflammation. Clinically, the hyperchylomicronaemic state can also interfere with laboratory assays (e.g., spuriously low amylase in lipaemic serum), so diagnosis should not rely solely on enzyme levels [2].

Mechanistically, the ‘Free Fatty Acid–FFA-toxicity’ model has important therapeutic implications. Hydrolysis of triglyceride-rich lipoproteins generates unbound FFAs that can exceed albumin binding capacity, trigger intracellular Ca^2+^ overload and mitochondrial dysfunction in acinar cells, injure the pancreatic microvascular endothelium, and promote capillary plugging and hypoperfusion. These events amplify local necroinflammation and systemic cytokine release, providing a rationale for rapid triglyceride lowering (to reduce further FFA generation) together with early, guideline-concordant supportive care [2,4].

## 5. Drivers of Severe Hypertriglyceridaemia and Pancreatitis Risk: Acquired and Genetic Contributors

Severe HTG potentially causing AP can be secondary (acquired) or primary genetic.

Secondary drivers commonly precipitate HTG-AP by pushing triglycerides above the chylomicronaemic threshold in individuals with underlying polygenic susceptibility [6]. The most frequent and clinically actionable contributors include uncontrolled diabetes (often with ketosis), excess alcohol intake, obesity and insulin resistance, diets high in refined carbohydrates and saturated fat, pregnancy (particularly the third trimester), chronic kidney disease and nephrotic syndrome, and untreated hypothyroidism [7,8].

Several medicines can also raise triglycerides or trigger pancreatitis through additional mechanisms, necessitating careful drug reconciliation [9,10].

In intensive care, propofol infusion can raise triglycerides and has been linked to pancreatitis; triglycerides should be monitored during prolonged or high-dose infusions [11,12,13].

Table 1 summarizes a pragmatic checklist of secondary drivers and typical clinical ‘red flags’. In practice, more than one secondary driver is often present, and their correction frequently yields the greatest absolute reductions in triglyceride [6,7,8].

Severe hypertriglyceridaemia with chylomicronaemia can reflect either familial chylomicronaemia syndrome (FCS) or multifactorial/persistent chylomicronaemia. FCS is a rare autosomal recessive disorder caused by biallelic loss-of-function variants in genes required for intravascular triglyceride hydrolysis (e.g., LPL, APOC2, APOA5, GPIHBP1, LMF1). It often presents in childhood or adolescence with recurrent abdominal pain, eruptive xanthomata, lipaemia retinalis, and recurrent pancreatitis despite strict dietary fat restriction [14]. By contrast, multifactorial or persistent chylomicronaemia is far more common and results from polygenic predisposition compounded by secondary drivers; triglycerides may fluctuate widely and often respond (at least partially) to correction of acquired contributors and conventional pharmacotherapy [14].

A recent joint National Lipid Association/American Society for Preventive Cardiology consensus proposes a pragmatic umbrella term, persistent chylomicronemia (PC), defined as triglycerides ≥1000 mg/dL in more than half of measurements, and emphasises phenotype-based risk stratification. Genetic FCS remains rare (~1–10 per million), whereas multifactorial chylomicronemia is far more common (~1 in 500). The statement also highlights ‘alarm’ features (e.g., recurrent triglyceride-induced pancreatitis, childhood pancreatitis, family history of triglyceride-induced pancreatitis, or markedly reduced post-heparin LPL activity) that identify patients whose pancreatitis risk approaches that of FCS and who may benefit from expedited access to intensive triglyceride lowering (including apoC-III–targeted therapies) [15].

Because access to emerging RNA-targeted therapies is often restricted to genetically confirmed or strongly suspected FCS, early differentiation is clinically important. Simple clinical clues include early onset, very low LDL-C/apoB, minimal response to fibrates/omega-3, and recurrent pancreatitis at comparatively stable triglyceride levels. Scoring systems (e.g., the FCS score) can support triage for genetic testing [16]. These practical differentiating features are summarized in Table 2.

## 6. Acute Management of HTG-AP

Initial management of HTG-AP follows general acute pancreatitis principles: early risk stratification, aggressive but judicious intravenous fluids, prompt analgesia, early enteral nutrition when feasible, and management of complications (organ failure, infection, necrosis) according to established guidelines. Triglyceride-specific treatment is typically considered when triglycerides are markedly elevated (commonly ≥11.3 mmol/L) and/or when chylomicronaemia is suspected to be contributory. In patients with concurrent diabetic decompensation, intravenous insulin addresses both hyperglycaemia and triglyceride-rich lipoprotein clearance through the upregulation of lipoprotein lipase [1,2,3,4,5,6].

Evidence supporting therapeutic plasma exchange (TPE) is mixed. In a randomized trial comparing TPE with intravenous insulin, TPE produced a numerically faster decline in triglycerides but did not improve clinical outcomes such as organ failure or intensive care unit (ICU) length of stay [17]. In a large contemporary observational analysis, early plasmapheresis was not associated with improved in-hospital outcomes after adjustment for baseline disease severity [18]. A meta-analysis of 15 studies (1 randomized, 2 prospective case-control, and 12 retrospective cohort studies) involving 909 patients with HTG-AP concluded that insulin-treatment was less effective than TPE in reducing triglyceride levels (Δ-TG) at 24 h (WMD, −666; 95%CI, −1130 to −201; *p* = 0.005), 48 h (WMD, −672; 95%CI, −1233 to −111), and by day 7 (WMD, −385; 95%CI, −711 to −60; *p* = 0.02), even if insulin-treatment was associated with fewer adverse events and lower costs [19]. Accordingly, current apheresis guidance reserves TPE for carefully selected, high-risk patients (e.g., refractory or extremely high triglycerides with evolving organ dysfunction), ideally within a multidisciplinary framework [20].

**Figure 1:**
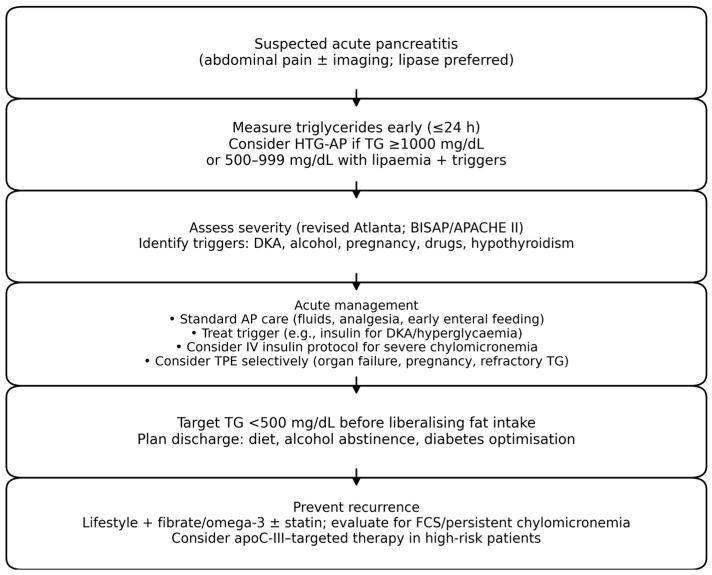
Pragmatic diagnostic and management algorithm for suspected hypertriglyceridaemia-associated acute pancreatitis (HTG-AP). Abbreviations: TG, triglycerides; TPE, therapeutic plasma exchange; FCS, familial chylomicronaemia syndrome.

Key practical points include: (i) measure triglycerides early (ideally at presentation, before prolonged fasting); (ii) avoid intravenous lipid emulsions unless clinically necessary; (iii) consider stopping triglyceride-raising medications; and (iv) aim for rapid reduction to <5.6 mmol/L (<500 mg/dL) in high-risk scenarios, recognizing that targets and timelines vary across centers [6,7,8,19]. A pragmatic diagnostic and management pathway is illustrated in Figure 1.

## 7. Long-Term Prevention of Recurrence

Recurrent pancreatitis after an index HTG-AP episode is common and clinically meaningful, with prospective and retrospective cohorts reporting substantial readmission rates, particularly when post-discharge triglycerides remain elevated. Persistent hypertriglyceridaemia during follow-up, ongoing metabolic triggers (e.g., uncontrolled diabetes, obesity, alcohol exposure), and a higher comorbidity burden have been identified as key predictors of recurrence [21]. These data provide the clinical rationale for ongoing interventional trials, including the multicentre REDUCE randomised controlled trial protocol, which will test whether intensive triglyceride targets (<150 mg/dL) reduce recurrent HTG-AP compared with usual care (<500 mg/dL) [22].

Preventing recurrence requires a dual strategy: (i) sustained suppression of triglycerides below the chylomicronaemic range; and (ii) removal of precipitating ‘second hits’. After an HTG-AP episode, most experts target triglycerides <5.6 mmol/L (<500 mg/dL), and ideally <2.3 mmol/L (<200 mg/dL) when feasible, recognising that the achievable target depends on the underlying phenotype [6,7,8].

Lifestyle measures remain foundational: strict limitation of dietary fat (in particular in FCS patients, <10–15% of daily calories, typically ≤15–20 g/day of long-chain fat, to minimise chylomicron formation, eventually using medium-chain triglycerides (MCT) as calorie source), fat spreading evenly across meals/snacks and avoid “fat boluses” (single high-fat meals) to reduce acute TG spikes, complete avoidance of alcohol, reduction of refined carbohydrates (prioritising complex, high-fibre carbohydrates), avoidance of high-fructose foods/drinks (soft drinks/juices), increase of aerobic physical activity, weight loss, and optimisation of glycaemic control. In multifactorial hypertriglyceridemias, reducing simple sugars and achieving modest weight loss can lead to large reductions in triglyceride [23].

### 7.1. Conventional Pharmacotherapy

The most extensively studied lipid-lowering drugs do not produce a marked reduction in triglyceride levels, largely because patients with very high triglyceride levels are usually excluded from clinical trials evaluating these agents [24]. Fibrates are commonly used for severe hypertriglyceridaemia and can reduce triglycerides by 30–50% in multifactorial phenotypes [25], though the benefit could be limited in FCS [26]. High-dose prescription omega-3 fatty acids (e.g., icosapent ethyl or eicosapentaenoic/doxosahexaenoic fatty acids formulations, 4 g/day) can add a further 20–30% reduction [27]. Statins are not primary triglyceride-lowering agents but are essential for cardiovascular disease risk reduction in appropriate patients and may modestly lower triglycerides [28]; they are not associated with an increased risk of pancreatitis in meta-analyses of 16 randomized trials with 113.800 patients and may show a non-significant trend toward lower risk [29]. Ezetimibe can also mildly contribute to the plasma TG reduction, as well as bempedoic acid and proprotein convertase subtilisin/kexin type 9 (PCSK9) inhibitors [24].

### 7.2. Emerging and Recently Approved Therapies Targeting ApoC-III and ANGPTL3

Apolipoprotein C-III (apoC-III) is a key inhibitor of lipoprotein lipase and hepatic clearance of triglyceride-rich lipoproteins. Well-known TG-lowering drugs, such as prescription omega-3 fatty acids [30] and fibrates, significantly reduce TG plasma levels [31]. RNA-targeted therapies that suppress apoC-III can markedly lower triglycerides and are now clinically available for FCS in selected jurisdictions. In the APPROACH trial, the antisense oligonucleotide volanesorsen reduced triglycerides by 77% at 3 months, and 77% of participants achieved triglycerides < 750 mg/dL, but intensive platelet monitoring is required because of thrombocytopenia risk [32]. Newer agents aim to improve the efficacy–tolerability balance: in a phase 2b trial, olezarsen lowered triglycerides by 43.5% at 6 months (vs. 6.2% with placebo), and pancreatitis episodes were less frequent (11 with placebo vs. 1 in each active-dose group) [33]. In the PALISADE trial, the small interfering RNA plozasiran reduced triglycerides by 80.2% at 10 months (vs. 18.9% with placebo) and was associated with lower pancreatitis incidence (odds ratio 0.17) [34]. The regulatory status of these agents continues to evolve across regions.

ANGPTL3 inhibition represents an alternative pathway by enhancing lipoprotein lipase activity. In a phase 2 trial, evinacumab reduced triglycerides in some severe hypertriglyceridaemia phenotypes, though effects were attenuated in classic FCS. Further outcome-focused studies are warranted to define which genetic and clinical subgroups derive the greatest pancreatitis risk reduction [35].

Table 3 provides a concise overview of emerging and recently approved triglyceride-lowering therapies, including their mechanisms of action and current regulatory status.

## 8. A Special Situation: The Pregnancy-Associated Severe Hypertriglyceridaemias

Pregnancy-associated severe hypertriglyceridaemia warrants proactive monitoring and multidisciplinary management, particularly in women with prior HTG-AP or suspected FCS [43]. Similarly, diabetic ketoacidosis with severe hypertriglyceridaemia can present with abdominal pain and pancreatitis; insulin remains central to management [44]. In a retrospective series of 116 pregnancies among 49 women with chylomicronemia (20 FCS, 29 MCS), at least one acute pancreatitis episode occurred in 42% of women with FCS versus 10% with MCS, and pancreatitis complicated 17% versus 5% of pregnancies; prematurity was common (56% in FCS vs. 19% in MCS), whereas miscarriage and fetal growth restriction rates were similar between groups [45]. Case-based evidence also supports structured multidisciplinary pathways: a 2025 report and systematic review of 23 pregnancies treated with regular apheresis found a mean triglyceride reduction of 45.1% per session (highest with centrifugation–filtration plasmapheresis: 68.3 ± 7.2%), no maternal deaths, but pancreatitis still occurred in 17.4% (mean triglycerides at onset 45.20 ± 4.65 mmol/L) [46].

## 9. Discussion

Hypertriglyceridaemia-related pancreatitis is a relatively rare but dramatic clinical complication of severe triglyceride-rich lipoprotein dysmetabolism and, importantly, it is often preventable [47]. In particular, severe forms of primitive massive hypertriglyceridemia should be screened as well as secondary causes of hypertriglyceridemia [7].

After an index episode, recurrence can also frequently be avoided through phenotype-driven detection and sustained management of severe hypertriglyceridaemia, together with systematic removal of precipitating ‘second hits’ [48,49] (Table 4).

In clinical practice, this work-up should start with a structured search for primitive massive hypertriglyceridaemia—most importantly familial chylomicronaemia syndrome (FCS) due to biallelic variants affecting the LPL pathway—because these patients carry a high lifetime recurrence risk and may benefit from early specialist referral, genetic confirmation, and access to targeted RNA-based therapies [15,50]. In this context, scientific societies are validating clinical scores to more easily and early identify subjects potentially affected by FCS [51,52,53,54].

Conversely, most cases reflect multifactorial or persistent chylomicronaemia, where genetic susceptibility is amplified by ‘second hits’. The most frequent triggers include uncontrolled diabetes or insulin deficiency, excess alcohol intake, obesity and metabolic dysfunction, and pregnancy-related triglyceride surges—particularly in the third trimester—often requiring multidisciplinary management [6,7,8]. Other secondary drivers that should be actively screened include hypothyroidism, nephrotic syndrome, and chronic kidney disease [55], and iatrogenic lipid-raising exposures such as oral oestrogens, retinoids, glucocorticoids, atypical antipsychotics, HIV protease inhibitors, and continuous propofol infusion in critically ill patients [56,57]. Importantly, documenting and addressing these reversible contributors is central to long-term prevention: the same patient may transition between moderate hypertriglyceridaemia and extreme levels depending on metabolic control, medication changes, and dietary adherence, underscoring the need for repeated assessment over time rather than a single ‘snapshot’ measurement.

Across guideline statements and technical reviews, the key practical message is to treat both the ‘fuel’ (triglyceride-rich lipoproteins) and the ‘second hits’ that precipitate attacks [7,48]. Early supportive care remains central, while triglyceride-lowering interventions are adjunctive and should be individualized to severity, comorbidity, and local expertise [54]. Secondary contributors (alcohol, uncontrolled diabetes, obesity, pregnancy, renal dysfunction) [55] and triglyceride-raising medications should be systematically sought and corrected, and drug causality considered when the temporal relationship is compelling [56,57].

For acute triglyceride lowering in HTG-AP, the evidence base remains heterogeneous. In a small randomized comparison, therapeutic plasma exchange (TPE) achieved a faster early reduction in triglycerides than insulin infusion, but did not demonstrate consistent superiority for clinically meaningful outcomes [52]; similarly, observational cohorts and meta-analyses yield mixed results and are prone to confounding by indication [58,59,60,61,62,63,64,65]. More recent retrospective comparisons and outcome analyses suggest broadly comparable clinical outcomes between extracorporeal approaches, with rapid biochemical improvements but an uncertain effect on organ failure or mortality, reinforcing a selective, individualized use in severe or refractory presentations [66]. Therefore, most contemporary guidance supports insulin-based protocols as first-line triglyceride-lowering therapy, reserving TPE or hemofiltration for severe disease with organ failure, refractory or extreme hypertriglyceridaemia, or when insulin is contraindicated, preferably within experienced centres [20]. New studies are trying to identify new (probably) most effective TPE protocols to improve prognosis in HTG-AP patients [67]. For long-term prevention, triglyceride-modifying outcome trials and emerging apoC-III/ANGPTL3–targeted approaches help frame the achievable magnitude of triglyceride lowering and the residual pancreatitis risk, but HTG-AP–specific prospective outcome data are still limited [36,37,38,39,40,41,42]. On the other hand, it remains unclear whether optimizing triglyceride levels *per se* reduces the risk of recurrent pancreatitis compared with a more conservative approach. This question is being addressed in an ongoing clinical trial enrolling 256 Chinese participants with hypertriglyceridemia-associated acute pancreatitis, who are randomized to either intensive triglyceride-lowering therapy (targeting TG <150 mg/dL) or usual care (targeting TG < 500 mg/dL) [22]. Finally, validated acute pancreatitis severity scores and broad reviews remain useful for bedside prognostication and for aligning HTG-AP management with general acute pancreatitis pathways [68].

Despite substantial advances in phenotyping and TG-lowering options, major evidence gaps persist: most interventional studies in HTG-AP remain small, heterogeneous, and focused on biochemical endpoints rather than patient-centered outcomes (persistent organ failure, necrosis, mortality), limiting confidence in the incremental value of strategies such as TPE over intensive medical therapy. Moreover, clinically relevant thresholds and definitions (e.g., “HTG-AP” attribution, “persistent chylomicronaemia”) are not yet fully harmonised across guidelines and studies, complicating risk stratification and cross-cohort comparisons. Finally, while apoC-III–targeted agents provide the strongest pancreatitis-prevention signal in FCS, high-quality data are still needed to define generalizability, optimal sequencing, and cost-effective implementation in broader multifactorial chylomicronaemia populations.

## 10. Conclusions

HTG-AP is a clinically important and often preventable cause of acute pancreatitis and recurrence. Risk is driven by chylomicronaemia and modulated by secondary triggers; systematic evaluation and correction of acquired contributors are therefore essential. Distinguishing FCS from multifactorial/persistent chylomicronaemia guides genetic testing, specialist referral, and access to emerging therapies. While supportive care remains the cornerstone of acute management, selected patients may benefit from rapid triglyceride-lowering using insulin-based regimens, with TPE reserved for severe or refractory cases. RNA-targeted therapies against apoC-III and ANGPTL3 pathway modulation represent a step change for the highest-risk phenotypes and may substantially reduce pancreatitis burden when integrated into comprehensive prevention programs.

## Figures and Tables

**Table 1:** Secondary drivers of severe hypertriglyceridaemia relevant to HTG-AP.

Driver	Examples, Mechanisms, and Practical Notes
Glycaemic decompensation	Uncontrolled type 2 diabetes, diabetic ketoacidosis, hyperosmolar state; insulin deficiency increases VLDL production and reduces lipoprotein lipase activity; treat aggressively (insulin, fluids), as triglycerides often fall rapidly.
Alcohol	Increases hepatic VLDL production and can directly injure the pancreas; binge patterns are particularly relevant; counsel on abstinence, especially after an HTG-AP episode.
Dietary excess/obesity	High refined carbohydrates and saturated fat amplify VLDL output; weight loss and carbohydrate restriction can yield substantial triglyceride reductions.
Pregnancy	Physiological hypertriglyceridaemia is accentuated in women with genetic predisposition; monitor closely in the third trimester; management may require specialist input and, rarely, apheresis.
Hypothyroidism	Reduces lipid clearance; screen with TSH in severe hypertriglyceridaemia and treat when present.
Renal disease/nephrotic syndrome	Impaired clearance of triglyceride-rich lipoproteins; consider in unexplained or refractory cases.
Medications	Examples include oestrogens, isotretinoin, certain antipsychotics, protease inhibitors, systemic corticosteroids, propofol, and others; switch or deprescribe where feasible.
Other	Sepsis and systemic inflammation, autoimmune disease activity, and rare endocrine disorders can contribute; manage underlying conditions.

Abbreviations: HTG-AP, hypertriglyceridaemia-associated acute pancreatitis; VLDL, very-low-density lipoprotein; TSH, thyroid-stimulating hormone.

**Table 2:** Practical features distinguishing FCS from multifactorial/persistent chylomicronaemia in the setting of severe hypertriglyceridaemia.

Feature	FCS (Monogenic)	Multifactorial/Persistent Chylomicronaemia
Typical onset	Childhood/adolescence	Adulthood (often with metabolic syndrome)
Triglycerides	Usually persistently very high; often >20–30 mmol/L	Variable; may spike with ‘second hits’
ApoB/LDL-C	Often very low (few VLDL/LDL particles)	Often normal/high (VLDL overproduction common)
Clinical features	Eruptive xanthomata, lipaemia retinalis, recurrent abdominal pain, frequent pancreatitis	Metabolic comorbidities; pancreatitis risk increases with severe peaks
Response to fibrates/omega-3	Limited	Often partial
Genetics	Biallelic loss-of-function variants in LPL pathway genes	Polygenic susceptibility ± heterozygous rare variants
Management emphasis	Very-low-fat diet lifelong; consider apoC-III/siRNA therapies when available	Correct secondary factors; pharmacotherapy (fibrates, omega-3, statins if indicated); consider emerging therapies for persistent high-risk cases

Abbreviations: FCS, familial chylomicronaemia syndrome; LDL-C, low-density lipoprotein cholesterol; VLDL, very-low-density lipoprotein.

**Table 3:** Novel triglyceride-lowering therapies relevant to HTG-AP: mechanism of action, approval status, and key evidence.

Agent (Trade Name)	Target/Modality	Key Population/Trial	TG Reduction	Approval Status (January 2026)	Key References
Volanesorsen (Waylivra)	ApoC-III antisense oligonucleotide (ASO)	FCS (APPROACH)	~77% at 3 months	EU/UK: conditionally authorized for FCS; requires platelet monitoring	[36,37]
Olezarsen (Tryngolza)	ApoC-III GalNAc-conjugated ASO	FCS (phase 2b/pivotal trial)	43.5% at 6 months	FDA-approved for FCS (Dec 2024); EMA authorized (2025)	[38,39]
Plozasiran	ApoC-III GalNAc-siRNA	Persistent chylomicronemia/PALISADE	80.2% at 10 months	Investigational/late-stage development (region-dependent)	[40]
Evinacumab (Evkeeza)	ANGPTL3 monoclonal antibody	Severe HTG (phase 2)	Variable; attenuated in classic FCS	Approved for HoFH (not specifically for HTG-AP)	[41]
Vupanorsen	ANGPTL3 antisense oligonucleotide (ASO)	Mixed dyslipidaemia (TRANSLATE-TIMI 70)	TG lowering; development limited by hepatic steatosis	Development discontinued/not approved for HTG	[42]

**Table 4:** Hypertriglyceridemia-related pancreatitis: main points.

The pancreatitis risk associated with hypertriglyceridaemia is driven primarily by chylomicronaemia and rises markedly above 11.3 mmol/L (≈1000 mg/dL), especially during metabolic stress. Secondary contributors (alcohol, uncontrolled diabetes, obesity, pregnancy, renal disease, and selected medicines) often act as ‘second hits’ and should be systematically sought and corrected. Distinguishing familial chylomicronaemia syndrome from multifactorial/persistent chylomicronaemia guides intensity of prevention, genetic testing, and access to emerging therapies. Acute management prioritises standard pancreatitis care plus rapid triglyceride lowering in selected patients; therapeutic plasma exchange remains reserved for severe or refractory cases. Diet, Statins, Ezetimibe, Fibrates, Omega-3 fatty acids can reduce triglyceride levels and related cardiovascular risk. Apolipoprotein C-III–targeted therapies (antisense oligonucleotides and siRNA) and ANGPTL3 inhibition can achieve large triglyceride reductions in selected phenotypes (e.g., volanesorsen ~77%; olezarsen 43.5% at 6 months; plozasiran ~80% at 10 months) with fewer pancreatitis events reported in FCS trials, strengthening long-term recurrence prevention in the highest-risk phenotypes.

## Data Availability

Not applicable.
